# Mycobiome-Host Coevolution? The Mycobiome of Ancestral Human Populations Seems to Be Different and Less Diverse Than Those of Extant Native and Urban-Industrialized Populations

**DOI:** 10.3390/microorganisms10020459

**Published:** 2022-02-16

**Authors:** Jelissa Reynoso-García, Yvonne Narganes-Storde, Tasha M. Santiago-Rodriguez, Gary A. Toranzos

**Affiliations:** 1 Environmental Microbiology Laboratory, Biology Department, University of Puerto Rico, San Juan 00931, Puerto Rico; gary.toranzos@upr.edu; 2 Center for Archaeological Research, Río Piedras Campus, University of Puerto Rico, San Juan 00931, Puerto Rico; ivonne.narganes@upr.edu; 3 Diversigen, Inc., Houston, TX 77046, USA; trodriguez@diversigen.com

**Keywords:** ancient DNA, ancient mycobiome, paleomicrobiology

## Abstract

Few data exist on the human gut mycobiome in relation to lifestyle, ethnicity, and dietary habits. To understand the effect of these factors on the structure of the human gut mycobiome, we analyzed sequences belonging to two extinct pre-Columbian cultures inhabiting Puerto Rico (the Huecoid and Saladoid) and compared them to coprolite samples found in Mexico and Ötzi, the Iceman’s large intestine. Stool mycobiome samples from extant populations in Peru and urban cultures from the United States were also included. The ancient Puerto Rican cultures exhibited a lower fungal diversity in comparison to the extant populations. Dissimilarity distances showed that the Huecoid gut mycobiome resembled that from ancient Mexico. Fungal genera including *Aspergillus* spp., *Penicillium* spp., *Rasamsonia* spp., *Byssochlamys* spp., *Talaromyces* spp., *Blastomyces* spp., *Monascus* spp., and *Penicilliopsis* spp. were differentially abundant in the ancient and extant populations. Despite cultural differences, certain fungal taxa were present in all samples. These results suggest that culture and diet may impact the gut mycobiome and emphasize that modern lifestyles could be associated with the alteration of gut mycobiome diversity. The present study presents data on ancient and extant human gut mycobiomes in terms of lifestyle, ethnicity, and diet in the Americas.

## 1. Introduction

Humans have coevolved with their gut microbiome, which plays an essential role in human health and well-being. The human and other animal gut microbiomes may be affected by factors such as geography, lifestyle, genetics, environment, and diet [1,2,3,4,5]. In addition to bacteria, diversity in intestinal fungi (both transient as well as intrinsic, referred to as the mycobiome) is being revealed. Recent studies have shown that modern lifestyles may result in a decreased diversity of the bacteriome and may have an impact on metabolic and immune diseases [6,7,8]. However, there is an information gap on the impact modern lifestyles and ethnicity may have on the gut mycobiome composition.

The human gut mycobiome of Westernized cultures seems to be mainly composed of the genera *Saccharomyces*, *Malassezia*, and *Candida*, with the species *Saccharomyces cerevisiae, Malassezia restricta*, and *Candida albicans* dominating the human stool samples tested [9]. These fungal species have also shown to be persistent across time, suggesting that they may not be transient. This study also showed that the gut mycobiome usually exhibits a high degree of inter- and intra-subject variability. Gut fungal communities are also known to help maintain homeostasis and can directly influence host metabolism, and indirectly via alterations to bacterial community composition [10,11,12,13,14]. To understand the composition and effect of modern lifestyles and ethnicity on the human gut mycobiome, it is important to understand it through part of human evolutionary history. However, studies on the ancient human gut mycobiome significantly lag when compared to those on the ancient bacterial and viral components [15,16,17,18,19,20].

Coprolites are contributing valuable information on the gut microbiome of ancestral populations. In addition, coprolites provide insights to understand how humans and the gut microbiome coevolved in response to changes in environment, culture, and diet. Coprolites from two pre-Columbian cultures, the Huecoid and Saladoid, recovered in Puerto Rico have previously been characterized by our group to determine the gut microbiota [17,18], viral communities [19] and parasite composition [21], to further support archaeological evidence suggesting that two pre-Columbian populations, the Huecoid and Saladoid, were two different cultures. Prior the 1980s, most of the archaeological evidence suggested that the Huecoid and Saladoid were the same culture. One of the main standing hypotheses is that the Saladoid culture migrated from Venezuela during the last centuries of the pre-Christian era and the first of the Christian era, whereas the Huecoid culture were an earlier migration of pottery-making horticulturalists. Microbiome evidence by our group supported the archaeological findings of the Saladoid and Huecoid being different cultures [17,18,19,21]; however, the gut mycobiomes of these cultures have not been studied and compared to present and extant gut mycobiomes.

In the present study, we analyzed coprolites of the Huecoid and Saladoid cultures to determine their fecal gut mycobiome. To better understand how the gut mycobiome is impacted by modern lifestyles and human adaptation to different environments, the mycobiome from coprolites from the Huecoid and Saladoid cultures were compared to those obtained from Mexican coprolites, the large intestine content from Ötzi, the Iceman, as well as fecal samples from extant native populations of Peru (Tunapuco and Matses) and urban populations in the United States (US). The Matses are hunter-gatherers from the Amazon with limited access to medical care and have a diet composed of food obtained from the environment. The Tunapuco, on the other hand, are agriculturalists from the Andes that practice small-scale agriculture, and animal domestication. In contrast, the United States individuals have a Westernized lifestyle, with access to medical care and higher sanitation standards. Including these populations in the analysis allowed us to determine the possible impact(s) of modern lifestyles, ethnicity, diet, and geography on gut mycobiome composition and diversity. Therefore, the main aim of the present study was to determine the gut mycobiome composition of the Huecoid and Saladoid cultures in comparison to coprolites from Mexico, intestinal content from Ötzi, stool samples from extant native populations from Peru, and urbanized populations from the United States. We found that the α-diversity as well as the composition and structure distinguished the ancient from extant populations.

## 2. Materials and Methods

### 2.1. Study Site and Sample Collection

In the present study, we studied coprolites from the Huecoid (*n* = 4) and Saladoid (*n* = 5) cultures from La Hueca, Sorcé, an archeological settlement in Vieques, an island situated in the southeast of Puerto Rico (18°05′56″ Latitude North and 65°29′34″ Longitude West). The coprolites were recovered during an excavation by archeologists Chanlatte and Narganes and were stored in the Center for Archeological Research of the University of Puerto Rico. The geographical distance between the Huecoid and Saladoid archeological deposits were 15–20 km. Because excavations were conducted in a private property, no permissions were required except for the owner’s authorization. The age of the coprolites was determined using radiocarbon dating of shells and charcoal associated with the samples [22]. Coprolites were radiocarbon dated at Teledyne Isotopes (Westwood, NJ, USA) and BETA Analytic, Inc. (Miami, FL, USA) using standard protocols. The Huecoid coprolites were radiocarbon dated from 245 to 600 AD, whereas the Saladoid coprolites dated from 230 to 395 AD. The semi-arid climate in Sorcé, Vieques provided favorable conditions for the preservation of these coprolites and previous studies of coprolites from this archeological midden have shown that the samples have exhibited a well-preserved fecal microbiota [19,21].

### 2.2. Microbial DNA Isolation and Contamination Control

Methods for DNA extraction and sequencing were performed as described [19]. In brief, nine coprolites belonging to Huecoid (*n* = 4) and Saladoid (*n* = 5) cultures were processed in a class II biosafety cabinet exclusive for ancient DNA using strict protocols and control required for ancient DNA studies (i.e., protective clothes and sterilized equipment). The class II biosafety cabinet was cleaned with 70% ethanol and UV-light decontaminated for 30 min prior and after use. To eliminate contamination with environmental DNA, the surface of the coprolites was removed using a sterile scalpel, thus only the core of the coprolites was used for analyses. The coprolites’ cores were ground into a fine powder using a sterile mortar and pestle and moisten overnight in sterile C1 buffer at 4 °C. DNA was extracted from the Huecoid and Saladoid coprolites’ cores using PowerSoil DNA Extraction Kit (Mo Bio Laboratories, Carlsbad, CA, USA) following manufacturer’s recommendations with some modifications in the final membrane wash step. The samples were then pooled to one composite for each culture using a standard glycogen precipitation protocol due to low DNA yields.

### 2.3. Library Preparation and Shotgun Metagenomics Sequencing

DNA concentrations were measured using Qubit^®^ dsDNA High Sensitivity Assay Kit (Life Technologies, Carlsbad, CA, USA). Whole genome amplification (WGA) was performed using REPLI-g Midi kit (Qiagen, Valencia, CA, USA). Amplified DNA was purified using PowerClean DNAClean-Up Kit (MO BIO Laboratories, Carlsbad, CA, USA) and quantified on Qubit^®^ dsDNA High Sensitivity Assay Kit (Life Technologies). Libraries were prepared with Nextera DNA Sample preparation kit (Illumina, San Diego, CA, USA) according to the manufacturer’s instructions. The concentration of the libraries was evaluated using Qubit^®^ dsDNA High Sensitivity Assay Kit (Life Technologies, Carlsbad, CA, USA) Then, libraries were pooled in equimolar amounts and shotgun sequenced on an Illumina MiSeq paired-end platform [19].

### 2.4. Bioinformatics

#### 2.4.1. Read Processing and Quality Control

Paired Illumina reads were trimmed and filtered with Trim-galore using default parameters as implemented in metaWRAP Read_qc module (v1.2.4) [23]. Then, reads were aligned to the Homo sapiens reference genome (build Hg38) to remove human DNA sequences from the metagenomics datasets using BMTagger as implemented in metaWRAP Read_qc module. The quality of the raw reads was visualized with FastQC [24] and the resulting pre-processed reads were used for subsequent analysis.

#### 2.4.2. Comparison to Other Samples

Fastq files from published microbial metagenomics datasets were obtained from the NCBI Sequence Read Archive (SRA) database using the fasterq-dump command from the SRA Toolkit (v2.10.4). Apart from the coprolite sequences from the Huecoids and Saladoids, the public shotgun sequence datasets used in this work included: *n* = 3 coprolites from the Loma San Gabriel culture (Mexico) and an Iceman large intestine content sample downloaded from NCBI (BioProject ID PRJEB31971) [25]. As a means of comparison, a total of *n* = 24 extant stool from the Matses hunter-gatherers and *n* = 12 stool sequences from the agriculturalists Tunapuco (Peru), were downloaded from NCBI (BioProject ID PRJNA268964) [26]. Also included in the analyses were *n* = 28 stool sample sequences from the Human Microbiome Project (HMP) from extant United States individuals (BioProject ID PRJNA48479) [27]. These datasets had been sequenced on Illumina platforms and were processed with data produced in this study using the parameters previously described.

The urbanized population is composed of United States individuals living in metropolitan areas with a high number of individuals per geographic area. These individuals follow a western diet and have access to healthcare and sanitized environments. In contrast, the Matses hunter–gatherer population include individuals residing in isolated areas from the Peruvian Amazon. These hunter–gathers have limited access to medical care and their diet relies on food from the environment [26]. The Tunapuco agricultural population are those individuals living in the Andean highlands. Their diet consists of agricultural crops and domestic animals [26]. The pre-Columbian culture Loma San Gabriel inhabited the Rio Zape caves located in Durango, Mexico. These coprolites were found in the archeological site La Cueva de Los Muertos Chiquitos (1300 ± 100 BP). The subsistence of Loma San Gabriel culture was based on agriculture and hunting-gathering that varied among seasons [28,29]. The Iceman, commonly known as Ötzi, is a European Copper Age mummy preserved in an Italian Alpine glacier for more than 5300 years [25]. The Iceman was an Early European farmer from the Eastern Italian Alps [30,31,32].

#### 2.4.3. Taxonomic Profiling

Pre-processed fastq sequencing files were used for taxonomic classification with Kaiju (v1.5.0) [33] to assign reads to the lowest common ancestor [33,34] using the following parameters: −a greedy −10^0.05^ to filter matches through e-value. Kaiju classification was performed against a subset of the NCBI BLAST non-redundant reference database (argument -nr_euk) that include proteins from bacteria, archaea, viruses, fungi, and microbial eukaryotes (accessed on 25 May 2020).

#### 2.4.4. Data and Statistical Analysis

For general statistical analysis and data visualization, we used the R packages (v4.0.3): tidyverse (v1.3.1), cowplot (v1.1.1), picante (v1.8.2), vegan (v2.5.7), HMP (v 2.0.1), dendextend (v1.15.1), Microbiome (v1.12.0), ALDEx2 (1.22.0), ggplot2 (v3.3.5) and phyloseq (v1.34.0). At the genus level, the meta-taxonomic composition of the samples was done after removing taxa undetected more than three times in at least 20% of the samples to address genera with small means. The differences in relative abundance were assessed using the Xdc.sevsample function in the R HMP package to test for differences in the overall composition between the groups [35]. Samples were rarified to the minimum number of sequences in the samples to avoid potential bias associated with variation in sampling depth. Observed richness, Shannon, and Simpson [36] indices were determined using the phyloseq package [37], and used to estimate the gut mycobiome alpha-diversity across the groups. Kruskal–Wallis and Wilcoxon statistical tests were applied to evaluate statistical difference in the alpha diversity values, and to compare the inter-group variation in gut mycobiome composition. For beta-diversity, Permutational Multivariate Analysis of Variance (PERMANOVA) was used to evaluate statistical differences in the gut mycobiome structure of ethnic groups based on Aitchison distances index dissimilarity measure using the adonis function in the R phyloseq package [38]. The Aitchison distances were centered log ratio (clr)-transformed using the microbiome package in R and visualized onto two-dimensions using Principal Coordinate Analysis (PCoA) plots [39]. The dendrograms were constructed using the R vegan package to examine hierarchical clustering of the samples. Bray–Curtis dissimilarity measures were computed for all the samples and then Ward’s clustering algorithm was applied to assess sample clustering [40]. ANOVA-Like Differential Expression version 2 (ALDEx2) was applied to determine differentially abundant taxa across the groups. Significance differences were evaluated using the nonparametric Wilcoxon rank-sum test and *p*-values were adjusted for multiple comparison using the Benjamini–Hochberg method [41]. False discovery rate (FDR) of <0.05 was used as cut-off. The core mycobiomes were defined as those fungal taxa with an abundance >0.1% in at least 90% of the samples and were determined using the R Microbiome package.

## 3. Results

### 3.1. The Fecal Mycobiome of the Ancient Populations Is Less Diverse Than Those of Modern Populations

**Figure 1:**
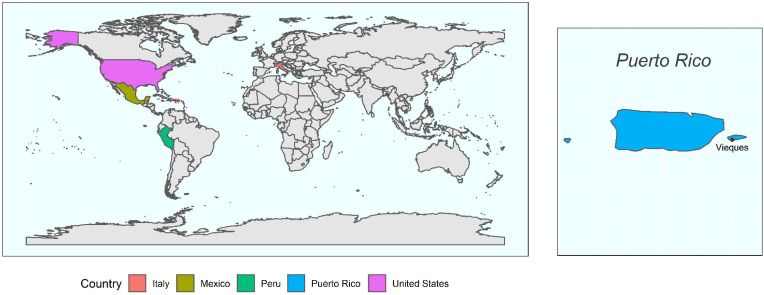
Geographic locations of the groups included in the present study. Puerto Rico is located between North and South America and is highlighted with a red square. Puerto Rico is magnified on the right side to show the municipality of Vieques, where coprolite samples were recovered.

Coprolites and extant fecal samples from six ethnic groups (Huecoid, Saladoid, Mexican, Matses, Tunapuco, and United States) distributed across four geographic areas (Figure 1) were analyzed using shotgun sequencing, resulting in 589,049 high quality sequences (including Ötzi the Iceman gut sample), with an average number of 8181 sequences per sample (ranging from 89 to 460,471).

We characterized the fecal mycobiome of the Huecoid and Saladoid cultures and compared the composition with those from previous studies, namely coprolites from Loma San Gabriel culture (Mexico) and a gut sample from Ötzi The Iceman [25], as well as extant stools from hunter–gatherers and agriculturalists from Peru [26] and urban individuals from United States [27].

**Figure 2:**
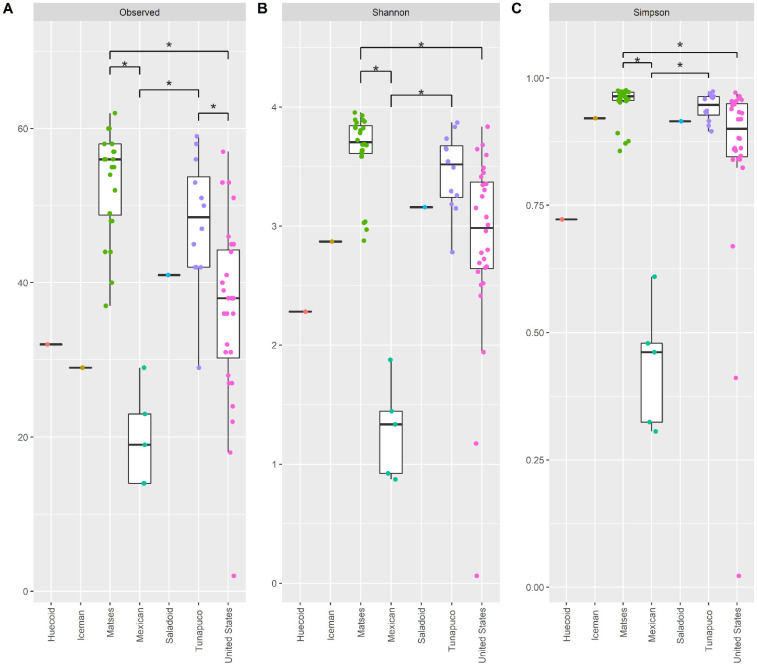
Alpha-diversity comparisons of the gut mycobiomes of each ethnic group. Analyses were performed at the genus-level. Boxplots show (**A**) observed richness, (**B**) Shannon, and (**C**) Simpson diversity of each ethnic group. Individual observations (dots) were colored according to ethnic group; * *p* < 0.05.

To elucidate the gut mycobiome’s α-diversity of the ethnic groups, we measured the observed number of species, as well as Shannon and Simpson indices within the samples using the fungal genera according to ethnicity (Figure 2) and culture (Figure S1). All the diversity measures showed that the gut mycobiome of the Mexican group is significantly less diverse than that of the Matses (Kruskal–Wallis rank sum test, *p*-value < 0.05) and the Tunapuco (Kruskal–Wallis rank sum test, *p*-value < 0.05); and in turn, the gut mycobiome of the United States individuals was significantly less diverse compared to the Matses (Kruskal–Wallis rank sum test, *p*-value < 0.05) (Figure 2). In addition, the observed richness of the United States gut mycobiome was significantly less diverse than that of the Tunapuco (Kruskal–Wallis rank sum test, *p*-value = 0.0241) (Figure 2A). No differences were detected between the gut mycobiome of the Matses and Tunapuco. The Huecoid and Mexican coprolites, and the Iceman gut sample had the lowest richness in the gut mycobiome. Nonetheless, the Iceman gut sample had a higher evenness, compared to the Huecoid and Mexican coprolites, which indicates a better distribution (relative abundance) of fungal taxa in the former. The Saladoid coprolites had a higher α-diversity compared to the other pre-Columbian cultures. On the other hand, the Matses extant stools showed the highest gut mycobiome α-diversity followed by the Tunapuco and United States extant stools, suggesting a greater richness and evenness of fungal genera in these samples. Based on culture, we found that the ancient populations (Huecoid, Saladoid and Mexican and The Iceman) exhibited a lower α-diversity in comparison to extant populations (Matses, Tunapuco and United States) (Mann–Whitney U-test, *p*-value < 0.001) (Figure S1). It has been previously reported that urban populations have a higher gut fungal diversity when compared to rural populations [42,43]. In contrast, the gut bacteriome of hunter–gatherers and agriculturalists has as higher bacterial richness compared to urban populations [1,26,44,45,46,47,48,49,50,51]. In this regard, we found that the Saladoid culture had the highest α-diversity. Moreover, we found that the United States individuals have a lower bacterial richness compared to Huecoids (Kruskal–Wallis; *p*-value = 0.0192), Saladoids (Kruskal–Wallis; *p*-value = 0.0209), Mexican (Kruskal–Wallis; *p*-value = 0.0040), Matses (Kruskal–Wallis; *p*-value < 0.001) and Tunapuco (Kruskal–Wallis *p*-value < 0.001) (Figure S2), which is consistent with previous studies.

### 3.2. Hierarchical Clustering Revealed a Certain Degree of Clustering among the Ancient and Modern Populations

**Figure 3:**
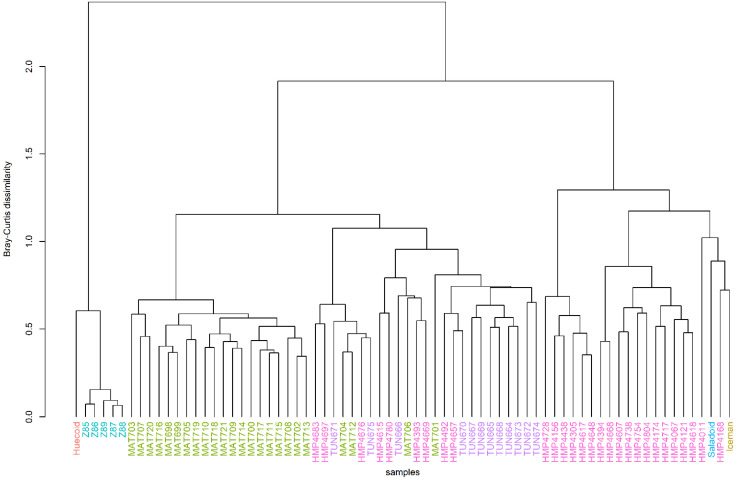
Hierarchical clustering. Hierarchical clustering of the Huecoid (coral/red), Saladoid (blue) and Mexican (turquoise) coprolites, and the Iceman gut sample (golden) as well as the Matses (green), Tunapuco (purple), and US (pink) extant stools using the Bray–Curtis dissimilarity measure.

To evaluate the extent to which samples from the ethnic groups clustered together, we performed a hierarchical clustering using the Bray–Curtis dissimilarity measure, which measures the difference in diversity between microbial communities. The Bray–Curtis dissimilarity is 0 when the samples have the same community composition, and 1 when the individuals share no fungal species. The hierarchical clustering of the gut mycobiome of the six ethnic groups showed some clustering of the samples according to ethnicity (Figure 3). However, several Tunapuco and United States extant stools samples showed distinctiveness in the gut community structure. In addition, we observed that the coprolites were more similar to each other than to extant fecal samples. The compositional dissimilarity between the coprolites and the extant stool samples led to separation of the samples.

### 3.3. Fungal Communities of the Ancient Populations Differ from Those of Modern Populations

**Figure 4:**
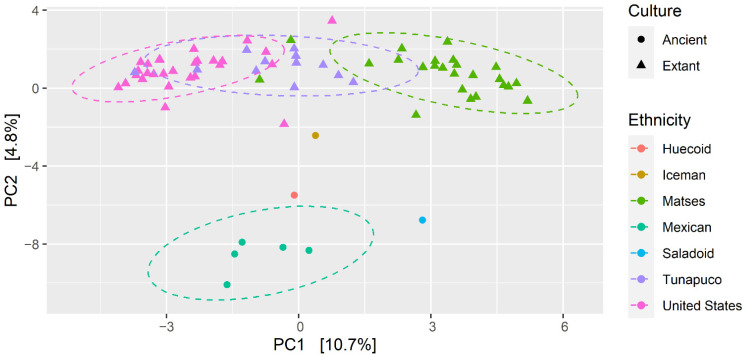
Beta-diversity comparisons of the gut mycobiomes of each ethnic group, principal coordinate analysis of Aitchison distances. The colors of the dots represent the different groups analyzed, whereas the symbols represent the ancient and extant cultures according to the legend. Symbols indicate whether cultures are ancient or extant.

**Figure 5:**
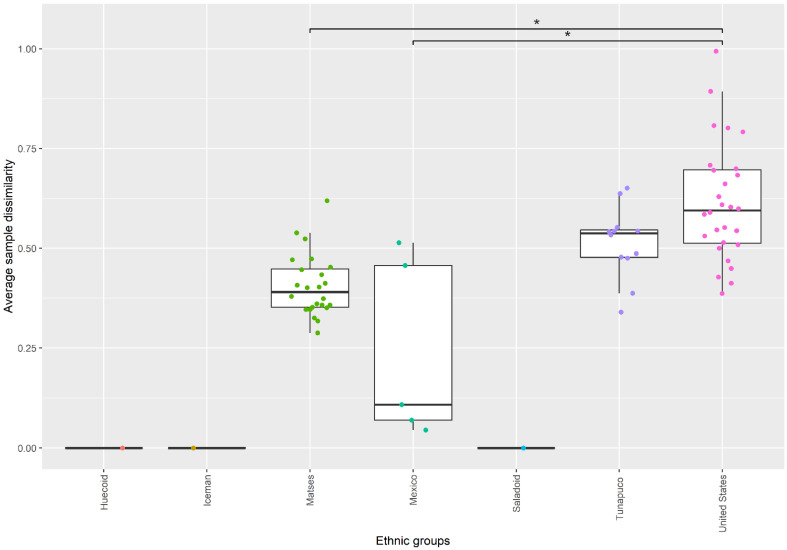
Mycobiome divergence across ethnic groups. Heterogeneity (inter-individual divergence) in community composition across the ethnic groups. Individual observations (dots) were colored according to ethnic group; * *p* < 0.05.

We calculated the microbial β-diversity, which are the differences in diversities across the samples, using the Aitchison distance (Euclidean distance of clr-transformed compositions). At the genus level, the PCoA ordination based on Aitchison distances showed a significant segregation between the ethnic groups (PERMANOVA, *p*-value = 0.001) (Figure 4), suggesting differences in the gut mycobiome composition and structure of these populations. However, the Huecoid and Saladoid were more similar to the Mexican coprolites than to the Matses and Tunapuco extant stool samples, which in turn were more similar to the United States extant stool samples. To compare the heterogeneity (inter-individual mycobiome divergence) in community composition across the ethnic groups, we quantified the average sample dissimilarity from the group mean. We found that United States extant stools samples had a more heterogenous gut mycobiome composition compared to the Mexican coprolites (Kruskal–Wallis rank sum test, *p*-value = 0.0032) and the Matses extant stools (Kruskal–Wallis rank sum test, *p*-value < 0.001) (Figure 5). In agreement with the higher inter-group variation observed in the United States extant stools, we observed higher dispersion of samples in this population (Figure 4). Moreover, an increased overall heterogeneity in community composition of extant populations in comparison to ancient populations was found (Mann–Whitney U-test, *p*-value = 0.01675) (Figure S3).

### 3.4. Metataxonomic Composition of the Samples Revealed Fungal Taxa That Differentiate Ancient and Modern Populations

**Figure 6:**
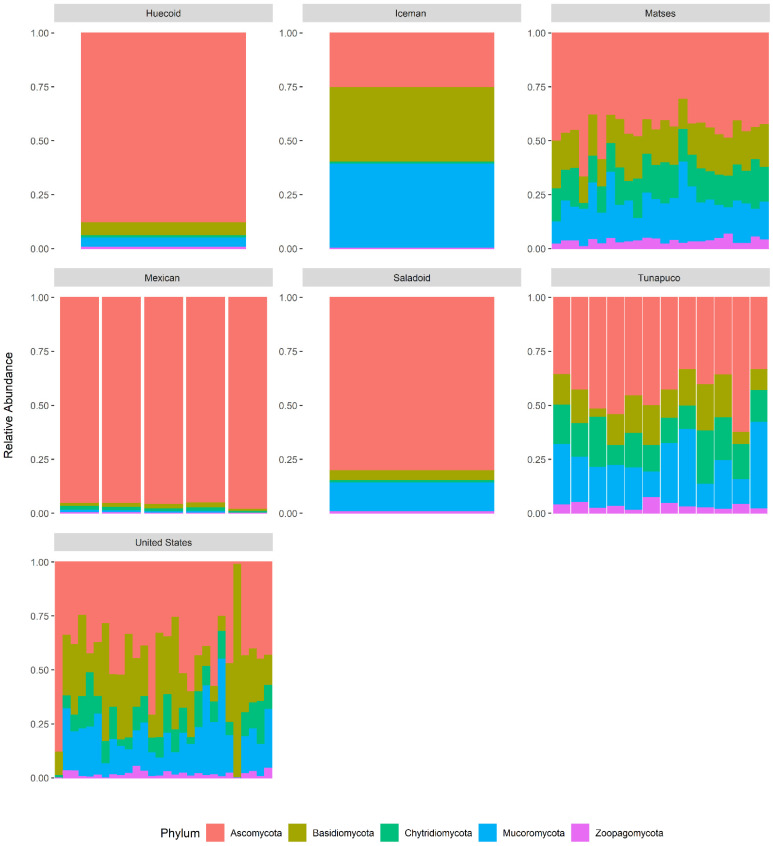
Gut mycobiome composition at the phylum level, relative abundance of all fungal phyla detected in the gut mycobiome of the ethnic groups.

To determine the fungal taxa that distinguished the ancient and modern populations, we compared the composition of the samples at the phylum and genus levels. In general, we identified five fungal phyla in the ancient and modern fecal mycobiomes (Figure 6). All the samples examined were dominated by Ascomycota, followed by Basidiomycota and Mucoromycota. The average relative abundance of Ascomycota was similar among the Huecoid (88%), Saladoid (80%), and Mexican (96%) coprolites and higher when compared to the Matses (45%), Tunapuco (44%), and United States (42%) extant stools, and the Iceman gut sample (25%). Nonetheless, the Basidiomycota phylum was enriched in the Matses (18%), Tunapuco (14%), and United States (27%) extant stools, and the Iceman gut samples (35%) in comparison with the Huecoid (6%), Saladoid (5%), and Mexican (2%) coprolites. In addition, the average relative abundance of Mucoromycota was higher in the Matses (19%), Tunapuco (23%), and United States (19%) extant feces, and the Iceman gut sample (39%) while lower in the Huecoid (5%), Saladoid (14%), and Mexican (1%) coprolites. The taxonomic composition of the samples also revealed marked differences in the relative abundance of the Chytridiomycota phylum, which was higher in the Matses (15%), Tunapuco (16%), and United States (10%) extant stools samples and rare in the Huecoid (0.5%), Saladoid (0.6%), and Mexican (1%) coprolites, and the Iceman gut sample (1%) (Figure 6).

**Figure 7:**
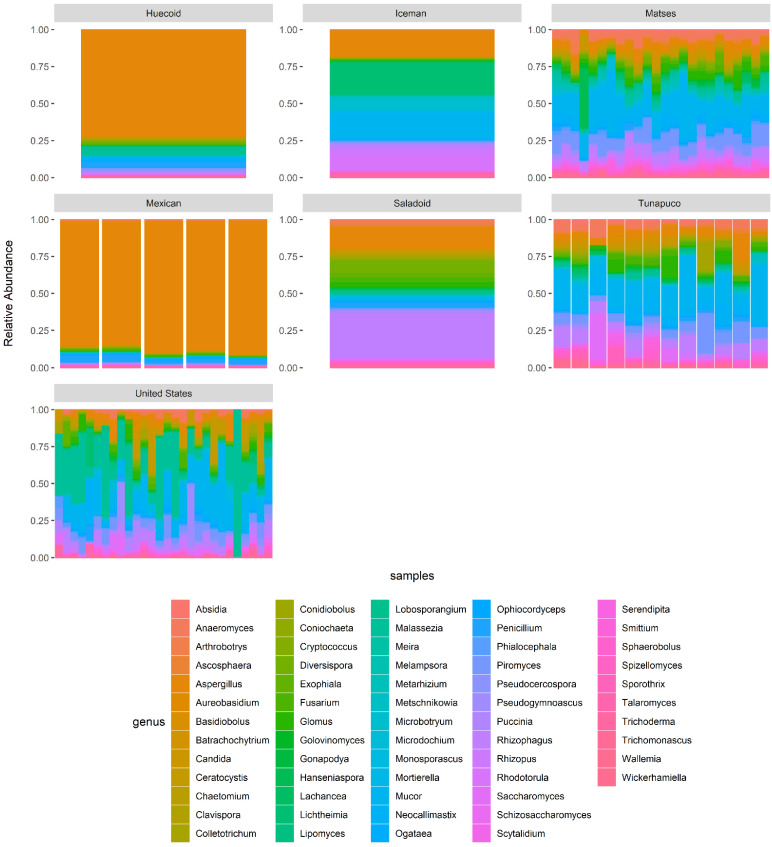
Gut mycobiome composition at the genus level, relative abundance of the fungal genera in the gut mycobiome of the ethnic groups (observed more than three times in at least 20% of the samples).

The major genera detected in the fecal mycobiome of the Huecoid culture were *Aspergillus* spp. (73% mean relative abundance), *Malassezia* spp. (6%), *Penicillium* spp. (5%), *Mucor* spp. (3%), and *Pseudocercospora* spp. (2%) (Figure 7). While the fecal mycobiome of the Saladoid culture was enriched in *Rhizophagus* spp. (31%), *Aspergillus* spp. (14%), *Diversispora* spp. (8%), *Glomus* spp. (5%), and *Penicillium* spp. (4%). In general, the most abundant fungal genera in the samples were *Aspergillus* spp., *Mucor* spp., *Rhizophagus* spp., *Malassezia* spp., and *Lichtheimia* spp. The overall mycobiome composition significantly differed among the group of samples (X_several sample test_, *p*-value < 0.001) (Figure 7). The mean relative abundance of the genus *Aspergillus* was higher in the Huecoid (74%), Mexican (65%), and Saladoid (6%) coprolites and the Iceman gut sample (18%) in comparison to the Matses (5%), Tunapuco (5%), and United States (5%) extant feces. In contrast, *Mucor* spp. were more abundant in the Matses (16%), Tunapuco (18%) and United States (15%) extant stools, and the Iceman gut sample compared to the Huecoid (3%), Saladoid (0.4%) and Mexican (0.3%) coprolites. Similarly, *Rhizophagus* spp. were more abundant in the extant stools of the Matses (5%), Tunapuco (7%) and United States (4%), but also in Saladoid coprolites (31%) whereas almost absent in the Huecoid (2%) and Mexican (0.1%) coprolites, and the Iceman gut sample (0.3%). Interestingly, extant stools from United States showed high proportions of *Malassezia* spp. (25%), whereas lower proportions were detected in the Huecoid (6%), Saladoid (2%) and Mexican (0.1%) coprolites, and the Iceman gut sample as well as the Matses (2%) and Tunapuco (1%) extant feces (Figure 7). The distribution of the sequences of each fungal taxa suggests that the Huecoid and Mexican pre-Columbian cultures have a low diversity in the gut fungal communities as these coprolites were dominated by *Aspergillus* spp. On the other hand, the relative abundance of the fungal genera detected in the Saladoid coprolites, and the Iceman gut sample as well as the extant stools from the Matses, Tunapuco, and the United States were well distributed, suggesting a more diverse gut ecosystem.

### 3.5. Differentially Abundant Fungal Genera in the Ascomycota phylum Were the Main Drivers of the Differences between the Ancient and Modern Mycobiomes

ALDEx2 was used to identify differentially abundant fungal genera associated to ancient and extant populations. The ALDEx2 method showed that *Aspergillus* spp., *Penicillium* spp., *Rasamsonia* spp., *Byssochlamys* spp., *Talaromyces* spp., *Blastomyces* spp., *Monascus* spp., and *Penicilliopsis* spp., distinguished the groups defined by culture (ancient and extant) (Table 1). All the fungal genera detected belong to the Ascomycota phylum, Pezizomycotina subphylum, Leotiomyceta clade, Eurotiomycetes class, and Eurotiomycetidae subclass. At the order level, we found genera belonging to the Eurotiales and Onygenales. The most abundant families were Aspergillaceae and Trichocomaceae followed by Thermoascaceae and Ajellomycetaceae.

### 3.6. Core Mycobiome Results Show That the Most Abundant Fungal Genera Were Shared among the Ancient and Modern Populations

**Figure 8:**
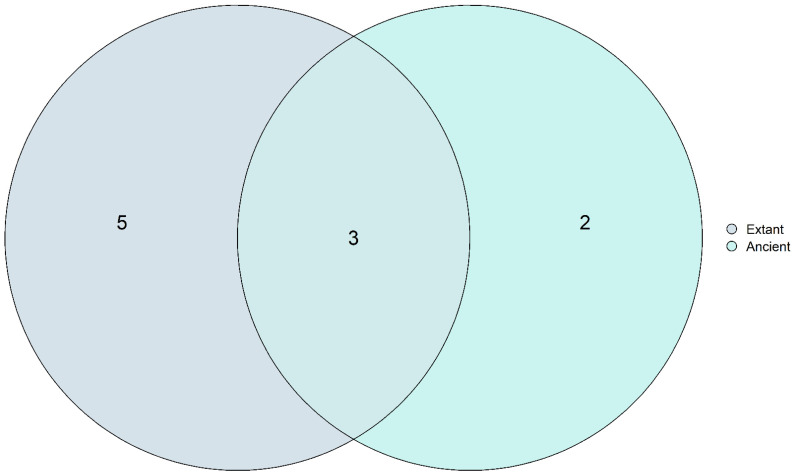
Core mycobiome. Total fungal genera detected with >0.1% relative abundance in at least 90% of the samples.

We considered the core mycobiome as the shared genera detected with >0.1% relative abundance in at least 90% of the samples [52]. The overall core mycobiome identified in all the ethnic groups were *Aspergillus* spp., *Fusarium* spp., *Malassezia* spp., *Mucor* spp., *Piromyces* spp., and *Rhizophagus* spp. (Figure 8). These genera were observed regardless of diet, culture, and lifestyle. Considering the ancient and extant populations, the core mycobiome of the Huecoid, Saladoid, and Mexican coprolites consisted of *Fusarium* spp., *Penicillium* spp., *Talaromyces* spp., *Mucor* spp., and *Aspergillus* spp., whereas *Anaeromyces* spp., *Neocallimastix* spp., *Fusarium* spp., *Rhizophagus* spp., *Malassezia* spp, *Mucor* spp., and *Aspergillus* spp. were the core fungi detected in the Matses, Tunapuco, and United States extant stools. Within these fungi, *Fusarium* spp., *Aspergillus* spp., and *Mucor* spp. were detected in both ancient and extant populations (Figure 8).

## 4. Discussion

The gut microbiome has immune and metabolic functions important to human health [53,54]. Modern lifestyles have the potential to impact the gut bacterial communities, resulting in a concomitant decrease in microbial diversity and an increase in diseases in the host. The study of the ancient microbiome preserved in archeological samples such as coprolites provide a window for characterizing these possible changes. Recent advances advocate for the consideration of the human microbiome while studying the evolution of humans. However, while more efforts have been made to incorporate the microbiome in the evolution of humans, most studies have focused on the bacterial communities, whilst fungal communities have been neglected. In the present communication, we considered a missing piece of the puzzle: the mycobiome. We analyzed the gut mycobiome in coprolites from pre-Columbian cultures (Huecoid and Saladoid) from Puerto Rico and compared them with Mexican coprolites and an Iceman gut sample. In addition, we included stool samples from extant native populations from Peru (Tunapuco and Matses), as well as urban populations from the United States. The information presented will contribute to a better understanding of the possible impacts of modern lifestyle (i.e., diet) and ethnicity on the gut mycobiome composition.

The gut mycobiome was used to successfully differentiate between extant and ancient cultures in the Americas and Europe (Ötzi the Iceman). These results are consistent with previous studies suggesting that the cultural traditions and dietary habits can exert a significant effect on a populations gut mycobiomes [9,18]. Overall, the Ascomycota was enriched in the Huecoid and Saladoid coprolites followed by Basidiomycota and Mucoromycota, consistent with previous studies showing that Ascomycota and Basidiomycota predominate as part of the human gut mycobiome [55,56]. The Ascomycota phylum has edible species as well as plant pathogens, and the presence of sequences suggests the consumption of Ascomycetes by these pre-Columbian cultures as well as the possible presence of phytopathogens in their diet [18]. Recently, Kabwe et al. reported differences in the gut mycobiomes of rural populations versus urban populations in Africa. The relative abundance of the phylum Ascomycota was higher in rural populations, whereas the phylum Basidiomycota was higher in urban populations [42]. Indeed, we found that the Huecoid, Saladoid, and Mexican coprolites had an increase in Ascomycota and a decrease in Basidiomycota compared to extant stools of the Matses, Tunapuco, United States, and the Iceman gut samples. The changes in the Ascomycota: Basidiomycota ratio throughout time suggests an adaptation of the human mycobiome in response to changes in our relationship with food, and, likely, the environment.

We also observed differences in the gut mycobiome of the ethnic groups at the genus taxonomic level. A higher prevalence of *Aspergillus* spp. was detected in the coprolites and the Iceman gut compared to the extant stools. While it is known that the genus *Aspergillus* is ubiquitous in the environment [57,58], the genus has also been previously reported in the human gut mycobiome [59,60,61]. *Aspergillus* spp. are capable of surviving the transit through the gastrointestinal tract; however, these species are presumed to be transient (allochthonous) due to their abundance in the environment and their introduction through diet, meaning that they may be acquired by consuming certain food items [62]. In fact, studies have revealed a higher abundance of *Aspergillus* spp. in vegetarians when compared to people with a carnivorous diet [62,63]. Therefore, the higher relative abundance of *Aspergillus* spp. in Huecoid, Saladoid, and Mexican coprolites might be associated with the contamination of a wide variety of food included in the diet of these pre-Columbian cultures or the consumption of fermented foods [64]. Indeed, archaeological evidence suggests that Saladoids consumed fermentable carbohydrates from root crops [65,66]. Similarly, the prevalence of *Penicillium* spp. was much higher in coprolites compared to extant stools. *Penicillium* spp. also causes food spoilage and the detection of *Penicillium* spp. in this study might be related to the ingestion of contaminated foods. These results are compatible with previous paleomicrobiological studies based on Terminal Restriction Fragment (T-RFLP) Analyses [18]. On the other hand, low levels of *Mucor* spp. were detected in the coprolites compared to the extant stools, and the Iceman gut sample. *Mucor* spp. are occasionally detected in feces of healthy humans [67,68]. Internal Transcriber Spacer (ITS)-based sequencing in obese and lean subjects has shown that *Mucor* spp. was more abundant in non-obese than in obese patients [68]. In addition, the low abundance of *Mucor* spp. in obese individuals was restored with loss weight, pointing to a possible association between diet and the gut mycobiome. The microbiota composition is sensitive to diverse perturbations, including dietary changes and the invasion of enteric pathogens [69].

Interestingly, the United States extant stools showed an increased relative abundance in *Malassezia* spp. as compared to the fecal samples from extant native communities from Peru as well as coprolites and the Iceman gut sample. *Malassezia* spp. have been described as a commensal of the skin and oral mycobiome [70,71]. Additionally, *Malassezia* spp. are frequently reported in the gut of adults [9,56,58,67,72,73] and infants [74]. It is possible that *Malassezia* spp. are acquired in early life during breastfeeding [75]. Nash et al. reported a high prevalence of *Malassezia* (i.e., *M. restricta*) in fecal samples from healthy volunteers of the HMP. Such data are consistent with our results. However, this yeast was rarely detected in samples from individuals with a western diet compared to vegetarian counterparts [62,63]. Similarly, *Malassezia* spp. was not detected [76] or was detected less consistently [77] in other studies. These differences could be due to differences in cohorts (diet and location) or methodologies. Therefore, whether *Malassezia* spp. survives transiently in the human gut needs further study.

The extant populations showed a more diverse gut mycobiome than the ancient populations. Melanized fungi are well-preserved [78,79]; however, taphonomic conditions could have contributed to the decomposition of chitin in the cell wall of some genera of fungi, which in turn could contribute to the decreased diversity observed in the coprolites and the Iceman gut sample. Nonetheless, our results are partially consistent with previous studies showing a higher gut fungal diversity in urban populations as compared to rural populations [42,43]. These results suggest that culture and dietary habits have the potential to impact the gut mycobiome diversity and emphasized that modern lifestyle could be associated with the alteration of the gut mycobiome α-diversity. Moreover, the effect of modern lifestyle on the gut mycobiome depends on ethnicity, which is in agreement with previous studies on the gut mycobiome from different cohorts [42,43,80].

The fungal community structure of the Huecoid and Saladoid coprolites was more similar to that of the Mexican coprolites than the Matses, Tunapuco, and United States extant stools, and the Iceman gut sample. Moreover, the Matses and Tunapuco extant feces were more similar to that of the United States. This suggests similarities in the relative abundance of fungal genera among the ancient and extant populations despite differences in traditional customs, geography, and genetics. One possible explanation is that the diet of the Huecoid and Saladoid cultures is more similar to that of ancient Mexican communities rather than extant native communities (Matses and Tunapuco) and urban-industrialized populations (United States). The Huecoid and Saladoid cultures were agriculturalists whose diet was mainly composed of root-crops and fruits [81,82], similar to the Mexican group included in the present study [29,83]. Regarding the extant populations, it has been shown that the gut bacterial microbiome β-diversity of urban populations are different from those of traditional communities [46,48]. These differences have been associated with several factors including a diet rich in fiber and complex carbohydrate in traditional populations and a diet rich in animal protein and sugars in urban populations. However, Jha et al. demonstrated that the gut bacterial microbiome of foraging populations that transitioned to farming were more similar to that of United States individuals [84]. In addition, the Hadza hunter–gatherers have reflected seasonal gut bacterial microbiomes related to seasonal availability of food [69]. Between seasons, the populations reflected differences in their bacterial microbiota and some taxa disappeared although reappeared when the seasons turned. Interestingly, when the bacterial taxa disappeared, the hunter–gathers’ bacterial microbiota were similar to those of industrialized microbiota [69]. These studies suggest that changes in dietary habits may shape the microbial community structure. The differences in the gut mycobiome of the Huecoid and Saladoid coprolites and the Tunapuco and Matses extant stools could be related to migratory events. The Huecoid and Saladoid cultures migrated from South America to the Caribbean regions, which suggests the possible role of environmental factors on the gut mycobiome. Differences in geography and climate may affect the fungi we are exposed, which in turn may impact the gut mycobiome composition [9,42]. In addition, industrialization also affects extant native societies as they are connected to the global commerce.

The Mexican coprolites and the Matses extant stools had the lowest inter-individual variation detected, whilst the United States extant stools showed the highest heterogeneity in community structure. Some studies have found lower inter-individual variation in the traditional populations than in urban-industrial populations [26,85]. Western populations have a diverse genetics backgrounds, cultural traditions and diet compared to non-western populations [48]. Likely, this heterogeneity in western populations led to selective pressures that increase the inter-individual variation observed in extant fecal samples from United States. Modernization is associated with increased hygiene and sanitation standards that could limit microbial transition among the individuals and increase the mycobiome dissimilarity among individuals [43].

The gut mycobiome appears to be less stable over time than the gut bacterial microbiome and is dependent on environmental factors and dietary habits. [9,63]. However, we identified fungal genera that may constitute an ancestral core mycobiome. *Aspergillus* spp., *Fusarium* spp., *Malassezia* spp., *Mucor* spp., *Piromyces* spp., and *Rhizophagus* spp. were detected in all the ethnic groups despite differences in lifestyle and ethnicity. *Aspergillus* spp., *Fusarium* spp., *Malassezia* spp., and *Mucor* spp. are frequently detected in the human gut mycobiome and are associated with diet [56,86]. There is evidence of a core bacterial microbiome that have coevolved with humans and play an important role in the host’s health [87,88]. However, information about the core mycobiome is scarce. The coprolite samples from the Huecoid, Saladoid and Mexican, and the Iceman gut sample shared a core mycobiome composed of *Fusarium* spp., *Penicillium* spp., *Talaromyces* spp., *Mucor* spp., and *Aspergillus* spp. Ancient cultures had a fiber-rich diet and complex carbohydrates, which could explain the detection of plant-associated fungi of the families Mucoraceae, Nectriaceae, and Aspergillaceae. For instance, *Fusarium* spp. are plant pathogens commonly detected in vegetarians [62,63]. On the other hand, the core fungi detected in the Matses, Tunapuco, and US extant fecal samples were *Anaeromyces* spp., *Neocallimastix* spp., *Fusarium* spp., *Rhizophagus* spp., *Malassezia* spp, *Mucor* spp., and *Aspergillus* spp. Previous studies have shown that gut fungi are usually transient and diet-associated suggesting that fungi might not colonize due to ecological niches or the human gut environment. However, the human gut has been considered the primary niche of few *Candida* spp. The genus *Candida* is commonly identified in the human gut [76,86,89]. Nonetheless, we detected low abundance of *Candida* spp., especially in ancient populations. These results are consistent with the low prevalence of *Candida* found in stool samples from an indigenous population living in a remote region of French Guiana [90].

Despite the limited sample size in our study due to the nature of coprolite samples, which in turn may influence the diversity comparisons, we observed differences across the ethnic groups. Although there were no statistical differences in all the groups, we observed a decreased diversity in the ancient populations compared to the extant populations. Future work should focus on various stages of human evolution to understand how modern lifestyle contributes to changes in the human mycobiome.

## 5. Conclusions

Gut mycobiomes in relation to diet and culture have not been thoroughly studied as the human gut bacteriomes and viromes have. The study of the ancestral mycobiome is essential to understand the effect of modern lifestyles on the gut mycobiome composition. Here, it was revealed that coprolites from the Huecoid and Saladoid pre-Columbian cultures as well as Mexican coprolites had a different taxonomic composition when compared to fecal samples from extant native communities from Peru, the Matses and Tunapuco, and the United States individuals. These differences may be a reflection of modern lifestyles and human adaptation to different environments. Overall, the α-diversity as well as the composition and structure distinguished the ancient from extant populations, with the pre-Columbian cultures harboring a lower total diversity and higher relative abundance of *Aspergillus* spp. whereas the extant populations were enriched for *Mucor* spp. and *Malassezia* spp. The gut mycobiome preserved in coprolites from pre-Columbian cultures may provide a baseline to better understand human holomicrobiome evolution.

## Figures and Tables

**Table 1:** ANOVA-like differential expression (ALDEx2). Fungal genera differentially abundant in ancient and extant cultures.

	diff.btw	diff.win	Effect	wi.ep	wi.eBH	Genus
1	−6.68239	2.648387	−2.44229	1.11 × 10^−5^	0.005524	*Aspergillus*
2	−4.12151	2.869157	−1.4792	2.81 × 10^−5^	0.00606	*Penicillium*
3	−4.1763	2.971508	−1.4539	3.07 × 10^−5^	0.006401	*Rasamsonia*
4	−4.44338	3.153649	−1.4055	3.57 × 10^−5^	0.006584	*Byssochlamys*
5	−2.98222	2.36935	−1.26018	0.000116	0.013226	*Talaromyces*
6	−3.63104	3.269716	−1.14088	0.000141	0.014606	*Blastomyces*
7	−3.90086	3.668713	−1.13541	0.000667	0.037039	*Monascus*
8	−4.02314	3.927779	−1.02516	0.000609	0.033089	*Penicilliopsis*

The diff.btw represents the median difference among the groups on a log base2 scale; diff.win constitutes the largest mean variation within ancient and extant groups; Effect designates the effect size of the difference (median of diff.btw/diff.win); wi.ep designates the expected value of the Wilcoxon test *p*-value; wi.eBH represents the adjusted *p*-values for multiple comparison using Benjamini–Hochberg.

## Data Availability

The data presented in this study are available on request from the corresponding author. The data are not publicly available as we are working on another communication. Publicly available datasets analyzed in this study are available under NCBI-SRA BioProjects IDs: PRJEB31971 (Mexican coprolites and The Iceman large intestine), PRJNA268964 (Matses and Tunapuco), and PRJNA48479 (United States).

## Supplementary Materials

The following are available online at https://www.mdpi.com/article/10.3390/microorganisms10020459/s1, Figure S1: Alpha-diversity comparisons of the gut mycobiomes of ancient and extant cultures, Figure S2: Alpha-diversity comparisons of the gut bacterial microbiomes of each ethnic group, Figure S3: Mycobiome divergence among ancient and modern cultures.
