# Supplementary figures and images for: Mycobiome-Host Coevolution? The Mycobiome of Ancestral Human Populations Seems to Be Different and Less Diverse Than Those of Extant Native and Urban-Industrialized Populations
Source: Microorganisms. 2022 Feb 16;10(2):459. doi: 10.3390/microorganisms10020459 (PMC8877467; doi:10.3390/microorganisms10020459)

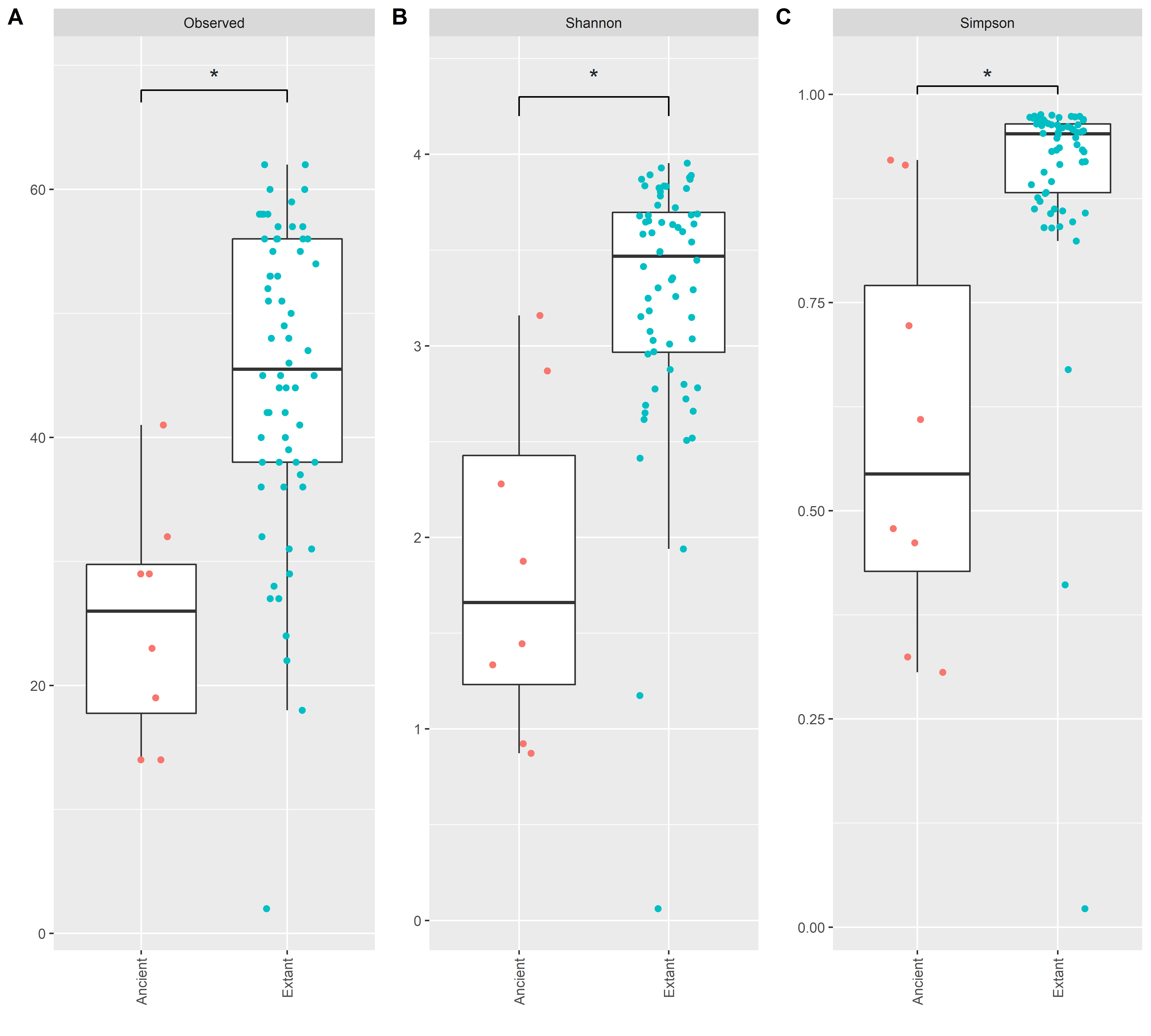

Supplement: Supplementary file 1 [file microorganisms-10-00459-s001.zip › Supplementary_material/FigS1.tif]

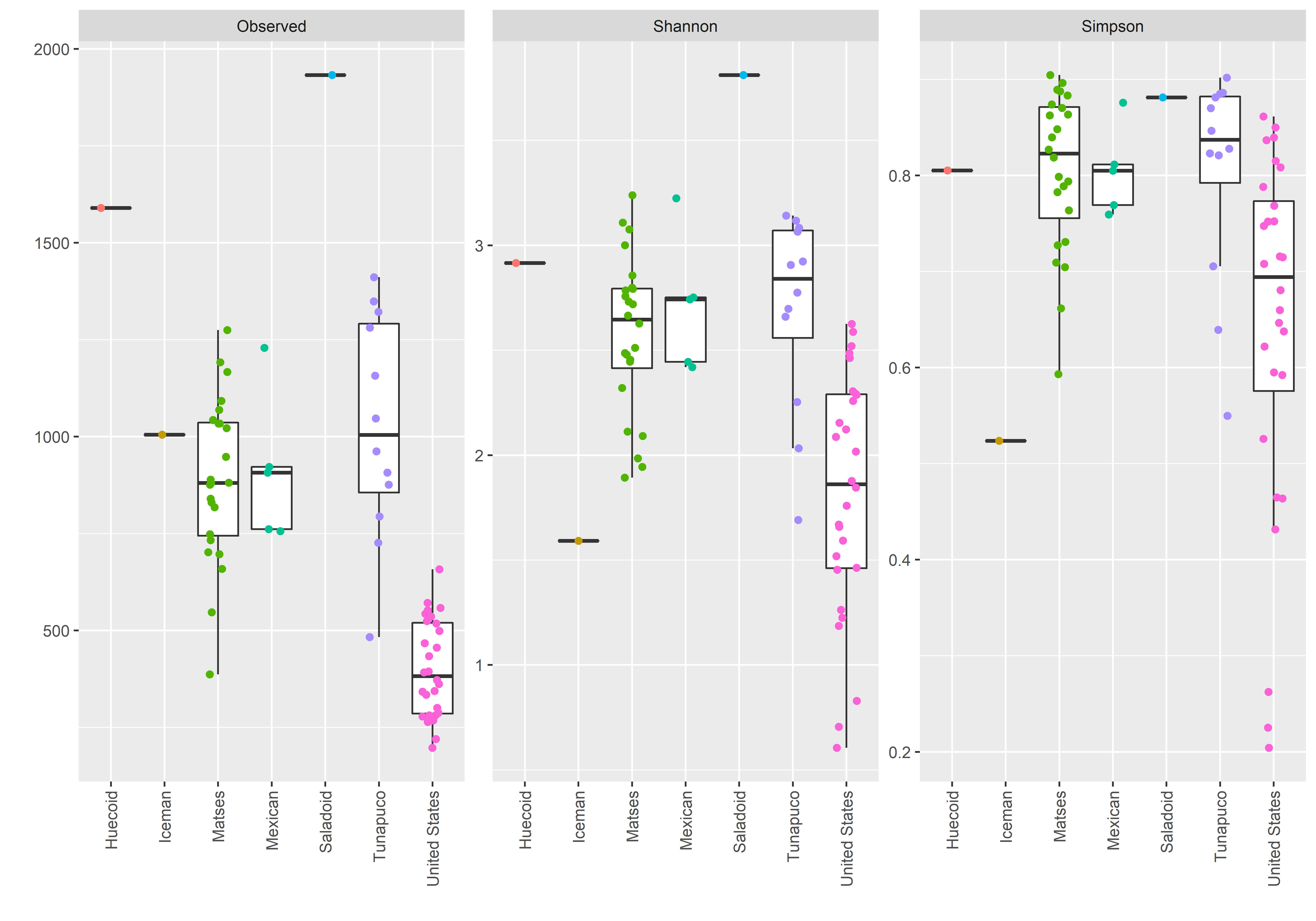

Supplement: Supplementary file 1 [file microorganisms-10-00459-s001.zip › Supplementary_material/FigS2.tif]

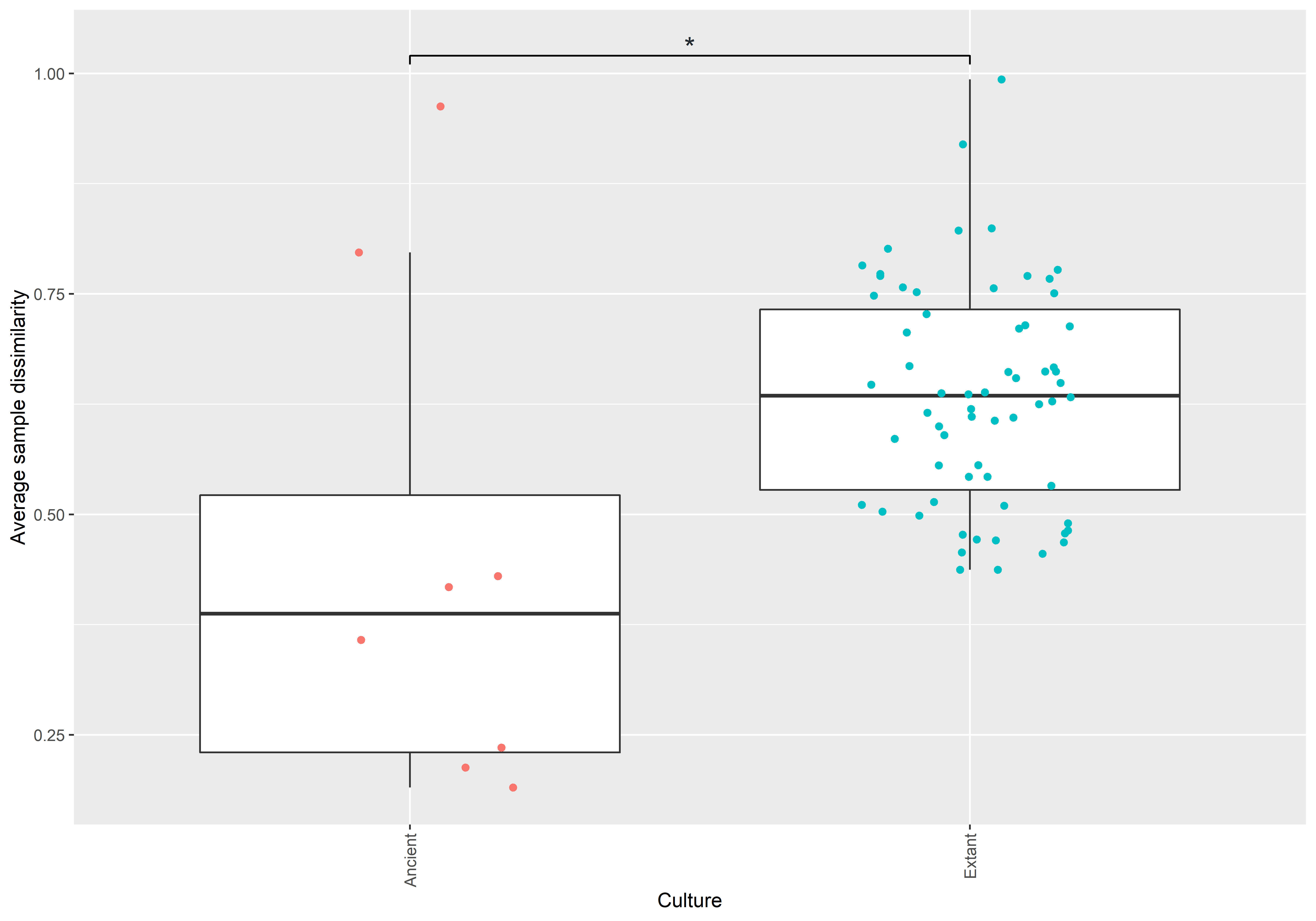

Supplement: Supplementary file 1 [file microorganisms-10-00459-s001.zip › Supplementary_material/FigS3.tif]
